# Slandering Physicians

**Published:** 1848-01

**Authors:** 


*Slandering Physicians.*—Uuder this caption, the Boston Medical and Surgical Journal publishes a correction, which we are truly glad to see.

A paragraph has been of late circulating through the newspaper and medical journals, in which it is stated that a surgeon of Lowell, Mass., after dressing the wound of a patient, and finding that he was to receive no fee therefor, tore off the dressings, and left the patient in that condition. The story seemed highly improbable on its face ; but as it was not only stated once, but reiterated by the newspaper in which it originated, we had reluctantly felt obliged to give it credence, and had prepared a notice of it, expressing sentiments of indignation, to which all our readers would have responded amen. It turns out that the allegation was utterly slanderous. Is there no legal redress for such grievances, to which, in some shape or other, physicians are constantly exposed? We do not, of course, intend to claim for the medical profession entire exemption from moral obliquities, but we do believe, that of the cases of criminal or immoral practices in which the perpetrators are designated *doctors*, the larger proportion are *pseudo-doctors*, and that in a large proportion of the remainder the offences are slanderously imputed.

We hope our friend of the N. Y. Annalist will not fail to make the correction in the instance referred to!

